# The MicroRNA-200 Family Is Upregulated in Endometrial Carcinoma

**DOI:** 10.1371/journal.pone.0022828

**Published:** 2011-08-29

**Authors:** Jaime Snowdon, Xiao Zhang, Tim Childs, Victor A. Tron, Harriet Feilotter

**Affiliations:** Department of Pathology and Molecular Medicine, Queen's University and Kingston General Hospital, Kingston, Canada; Bauer Research Foundation, United States of America

## Abstract

**Background:**

MicroRNAs (miRNAs, miRs) are small non-coding RNAs that negatively regulate gene expression at the post-transcriptional level. MicroRNAs are dysregulated in cancer and may play essential roles in tumorigenesis. Additionally, miRNAs have been shown to have prognostic and diagnostic value in certain types of cancer. The objective of this study was to identify dysregulated miRNAs in endometrioid endometrial adenocarcinoma (EEC) and the precursor lesion, complex atypical hyperplasia (CAH).

**Methodology:**

We compared the expression profiles of 723 human miRNAs from 14 cases of EEC, 10 cases of CAH, and 10 normal proliferative endometria controls using Agilent Human miRNA arrays following RNA extraction from formalin-fixed paraffin-embedded (FFPE) tissues. The expression of 4 dysregulated miRNAs was validated using real time reverse transcription-PCR.

**Results:**

Forty-three miRNAs were dysregulated in EEC and CAH compared to normal controls (p<0.05). The entire miR-200 family (miR-200a/b/c, miR-141, and miR-429) was up-regulated in cases of EEC.

**Conclusions:**

This information contributes to the candidate miRNA expression profile that has been generated for EEC and shows that certain miRNAs are dysregulated in the precursor lesion, CAH. These miRNAs in particular may play important roles in tumorigenesis. Examination of miRNAs that are consistently dysregulated in various studies of EEC, like the miR-200 family, will aid in the understanding of the role that miRNAs play in tumorigenesis in this tumour type.

## Introduction

Endometrial carcinoma is the most common gynecologic malignancy in Canada with an estimated 4700 new cases and 750 resultant deaths in 2011 [1]. Approximately 70 to 80 percent of newly diagnosed cases of endometrial cancer are endometrioid endometrial adenocarcinomas (EEC) which occur at a median age of 60. They are associated with chronic exposure to unopposed estrogen and are often preceded by complex atypical hyperplasia (CAH).

Recently, molecular studies have identified microsatellite instability and mutations in PTEN, K-ras, PIK3CA, and beta-catenin genes in cases of EEC [2], [3]. The identification of these molecular derangements can aid our understanding of EEC and can also lead to the discovery of novel targets for detection, prognosis, and prediction. Although these genetic alterations have been described, they are not universally present in all cases of EEC suggesting that other mutations or epigenetic alterations may also be important.

MicroRNAs (miRNAs) are a class of small RNAs (20–25 nucleotides in length) that are important regulatory molecules in plants, animals and viruses [4], [5]. Since their discovery [6]–[8], it has become clear that miRNAs regulate several key cellular processes including developmental timing [9], stem cell division [10]–[14] and apoptosis [15]–[17]. MicroRNAs likely influence these processes by post-transcriptional negative regulation of gene expression through binding to messenger RNA (mRNA) targets, causing mRNA cleavage, translational repression, or mRNA decay [5]. More than 50 percent of human miRNA genes are located at fragile sites or in regions known to be dysregulated by loss, amplification or breakage in cancer [18]. Using molecular techniques, dysregulated miRNAs have been identified in many human cancers including those of the lung, breast, testes, prostate, ovary, liver, pancreas, brain, colon, and in hematological malignancies like leukemia [19]. In some cases, the dysregulated miRNAs confer a “tumour signature” that can be exploited for diagnostic purposes. It has been proposed that overexpressed miRNAs may function as oncogenes while those that are underexpressed behave as tumour suppressor genes [19]. MicroRNA expression profiles are becoming useful tools in cancer diagnostics, screening and prognosis, and may play a future role in cancer therapeutics.

In this study, we used the Agilent microarray platform to screen for differentially expressed miRNAs in EEC as well as in the precursor lesion, CAH. We validated the expression of key miRNAs using real-time reverse transcription-PCR (real time RT-PCR). The aims of this study were to determine the panel of dysregulated miRNAs in EEC for further use in diagnostics and to investigate the potential role of miRNAs in this tumour.

## Methods

### Ethics Statement

This study was approved by the Queen's University Research Ethics Board and all participants signed a consent form prior to their surgical procedure.

### Samples

For discovery, formalin-fixed paraffin-embedded (FFPE) samples of CAH (n = 10), EEC (n = 14) and normal proliferative endometrium (n = 10) from hysterectomy specimens were identified in the Pathology Department at Kingston General Hospital (Ontario, Canada). For the biological validation studies, additional FFPE cases of CAH (n = 4) and EEC (n = 5) from endometrial biopsies or hysterectomy specimens were identified. Inclusion criteria for all cases included: (i) unambiguous histology and absence of mixed tumour types; (ii) sufficient viable tissue available for RNA extraction; (iii) absence of any treatment prior to surgery; and (iv) age of tissue block less than 7 years. The histology was reviewed by a gynecological pathologist (TJC) and areas on the block with sufficient tissue for analysis were marked. Two to 5 cores (0.6 mm) of tissue were obtained from the FFPE blocks for RNA extraction.

### RNA Extraction

Total RNA was extracted from the FFPE tissue cores using the Ambion RecoverAll Total Nucleic Acid Isolation Kit for FFPE tissues (Applied Biosystems/Ambion, Austin TX, USA) according to the manufacturer's instructions with a modified digestion time (5 hours). The quantity and quality of the total RNA was verified with the NanoDrop spectrophotometer (Thermo Fisher Scientific Incorporated, Wilmington DE, USA) according to the manufacturer's instructions.

### Labeling and Microarray

One hundred nanograms of total RNA was labeled using the Agilent miRNA Complete Labeling and Hybridization Kit (Agilent Technologies Incorporated, Santa Clara CA, USA) according to the manufacturer's instructions. The labeled RNA was hybridized to the Agilent Human miRNA Microarray (V2, Agilent) which contains probes for 723 mature human miRNAs. Arrays were scanned using an Agilent scanner and feature extracted using Agilent Feature Extraction Software, version 10.5.1.1.

### Analysis

Expression data were initially normalized to the 75^th^ percentile and then averaged among the groups using GeneSpring GX (Agilent) software. The Kruskall-Wallis test was used for comparisons among the groups and the Benjamini-Hochberg correction was applied to adjust for multiple comparisons. Unsupervised hierarchical clustering was performed using Genespring GX software.

### Target Prediction

MicroCosm Targets version 5 [20] was used to examine the gene targets of our miRNAs of interest.

### Real-time RT-PCR

To validate key microarray results, quantitative reverse transcription (RT) was performed using the TaqMan MicroRNA Reverse Transcription Kit (Applied Biosystems (ABI)), Foster City CA, USA) with ABI miRNA specific primers and primer kits on an Eppendorf Realplex Thermal Cycler (Mississauga ON, Canada). Specific kits used were as follows: miR-542-5p: ABI#002240; miR-200a: ABI#000502; miR-429: ABI#001024; miR-503: ABI#001048. Relative expression levels were calculated using the comparative Ct (2^−ΔΔCt^) method with U6 small nuclear RNA as the endogenous control. Samples were analyzed in triplicate. Technical validations were carried out using a subset of the original samples that were used in the discovery phase of the study (4 samples each of EEC and CAH and 3 normal controls). Biological validation was performed using additional FFPE cases of CAH (n = 4) and EEC (n = 5).

## Results

### Samples

Clinicopathologic information on the cases used for the microarray is included in Table 1. The carcinoma cases included predominantly stage I disease, with one case classified as stage II.

**Table 1:** Clinicopathologic characteristics of the cases used in the microarray.

Case Number	Age	Reason for Hysterectomy	FIGO Grade	FIGO Stage
Normal Controls				
99	38	Dysmenorrhia and menorrhagia		
57	37	Leiomyomata		
55	49	Uterine prolapse		
71	44	Ovarian cyst		
81	49	Leiomyomata		
80	40	Leiomyomata		
76	47	Uterine prolapse		
77	52	Uterine prolapse and menorrhagia		
64	28	Dysmenorrhea		
74	48	Ovarian cyst		
Atypical Hyperplasia Cases				
61	60			
98	59			
65	62			
97	61			
94	47			
62	63			
90	56			
73	59			
63	36			
79	59			
Endometrial Carcinoma Cases				
39	64		1	IA
16	64		1	IA
33	76		1	IA
37	68		1	IA
38	71		2	IB
10	56		2	IB
26	59		2	IA
29	47		2	IA
25	33		2	IA
48	49		2	IB
21	75		2	II
40	66		3	IB
34	67		3	1A
49	55		3	1A

FIGO = Federation Internationale de Gynecologie d'Obstetrique.

### Microarray Expression Profiles

As recommended by Shi et al [21], differentially expressed miRNAs were chosen based on a high fold change (at least 4-fold change in at least one group comparison) and a non-stringent *p* cutoff (*p*<0.05). This resulted in the identification of 43 miRNAs that differ significantly between EEC, CAH and normal controls (Tables 2 and 3).

**Table 2:** Differentially expressed microRNAs (p<0.05) showing decreased expression in EEC (fold change).

MicroRNA	CAH vs Control	EEC vs CAH	EEC vs Control
miR-100	−1.6	−3.4	−5.5
miR-10b*	2.2	−5.0	−2.3
miR-127-3p	−2.4	−1.8	−4.5
miR-152	−2.1	−2.2	−4.6
miR-199b-3p	−2.1	−2.4	−5.0
miR-199b-5p	−2.3	−3.2	−7.4
miR-23a*	−1.8	−2.2	−4.1
miR-370	1.4	−4.4	−3.2
miR-376a	−2.4	−2.8	−6.6
miR-376c	−2.1	−4.5	−9.6
miR-381	−2.1	−2.0	−4.2
miR-410	−2.4	−1.7	−4.2
miR-424	−4.1	−2.6	−10.7
miR-424*	−1.2	−4.0	−4.9
miR-431	2.2	−4.3	−1.9
miR-432	−1.9	−2.4	−4.6
*miR-503*	−8.6	−3.2	−27.3
miR-542-3p	−3.2	−2.1	−6.9
miR-542-5p	−2.0	−3.1	−6.1
miR-596	2.2	−4.4	−2.0
miR-610	1.9	−5.1	−2.7
miR-630	2.0	−5.2	−2.6
miR-632	1.9	−6.6	−3.4
miR-760	1.2	−5.4	−4.5

Down-regulated miRNAs are designated with a negative fold change. MicroRNAs which have similar expression changes in other studies of EEC are italicized.

**Table 3:** Differentially expressed microRNAs (p<0.05) showing increased expression in EEC (fold change).

MicroRNA	CAH vs Control	EEC vs CAH	EEC vs Control
***miR-141***	1.2	3.9	4.8
miR-146a	1.0	6.9	7.1
miR-18a	−2.9	4.4	1.5
***miR-200a***	1.2	4.9	6.1
***miR-200b***	1.1	5.9	6.6
miR-200b*	1.6	2.9	4.6
***miR-200c***	−1.0	4.3	4.2
*miR-203*	−1.1	6.2	5.4
*miR-205*	6.6	2.3	15.3
*miR-210*	1.2	5.7	6.7
miR-421	−1.5	4.0	2.8
***miR-429***	1.1	6.2	6.8
miR-516a-5p	4.4	−3.6	1.2
miR-605	−1.7	4.2	2.4
miR-614	4.5	−2.8	1.6
miR-9	1.3	4.0	5.1
miR-9*	2.3	3.5	8.2
miR-936	4.2	−3.0	1.4
*miR-96*	−1.1	4.3	3.8

Down-regulated miRNAs are designated with a negative fold change. MicroRNAs which have similar expression changes in other studies of EEC are italicized and the miR-200 family miRNAs are bolded.

### Hierarchical Clustering

**Figure 1:**
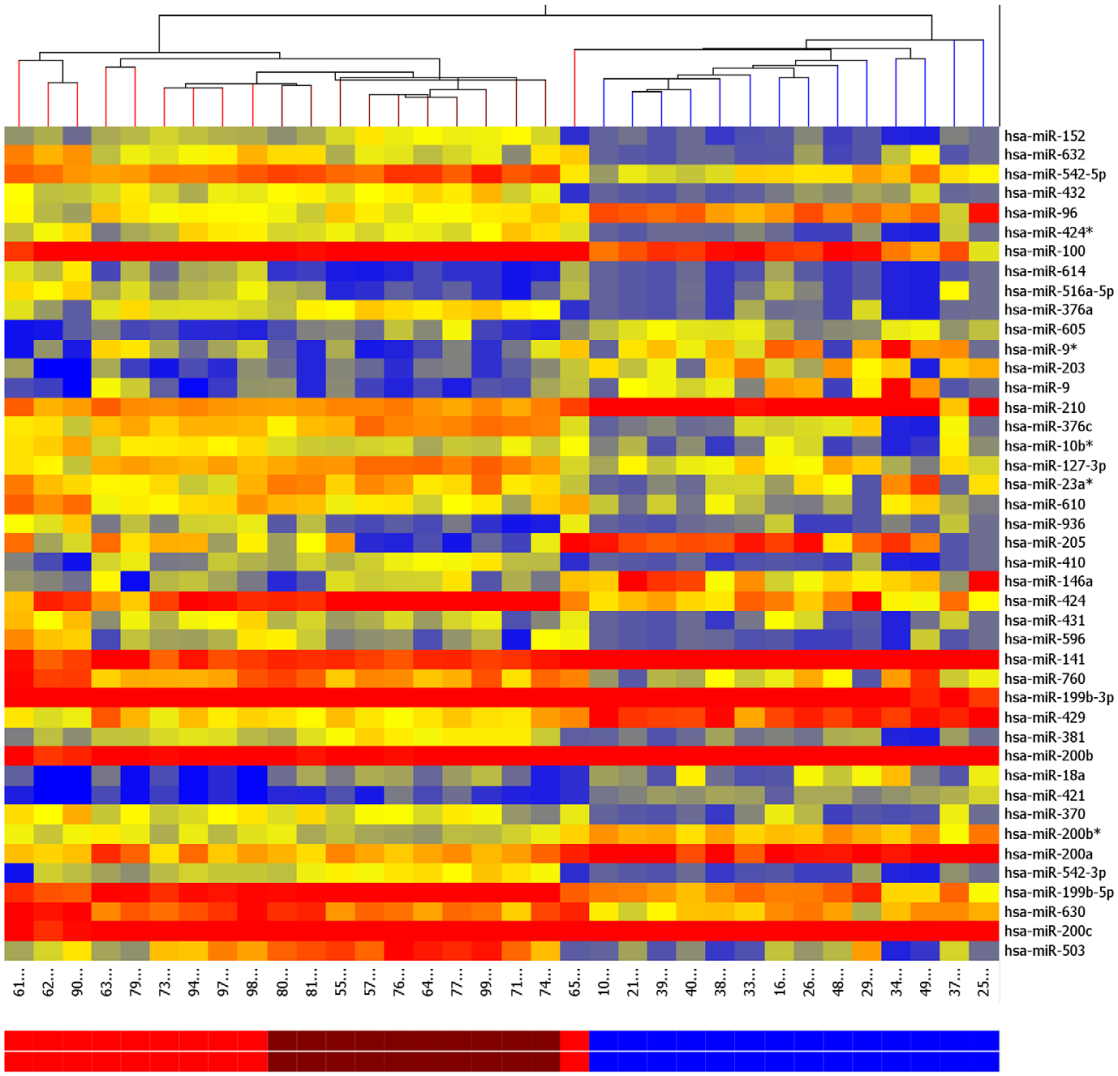
Unsupervised hierarchical clustering of normal endometrium (brown), atypical hyperplasia (red) and EEC (blue). Samples are in columns and miRNAs are in rows.

Figure 1 illustrates the results of the unsupervised hierarchical clustering based on the expression of the significantly differentially expressed miRNAs listed in Tables 2 and 3.

### Real-time RT-PCR

Two separate validation steps were performed using real-time RT-PCR. The first step was a technical validation using samples from the original set characterized on the microarray. For the biological validation step, RNA was isolated from a new set of FFPE tissues as described above to increase the likelihood that the observed differences in miRNA expression profiles represent biologically significant changes.

**Figure 2:**
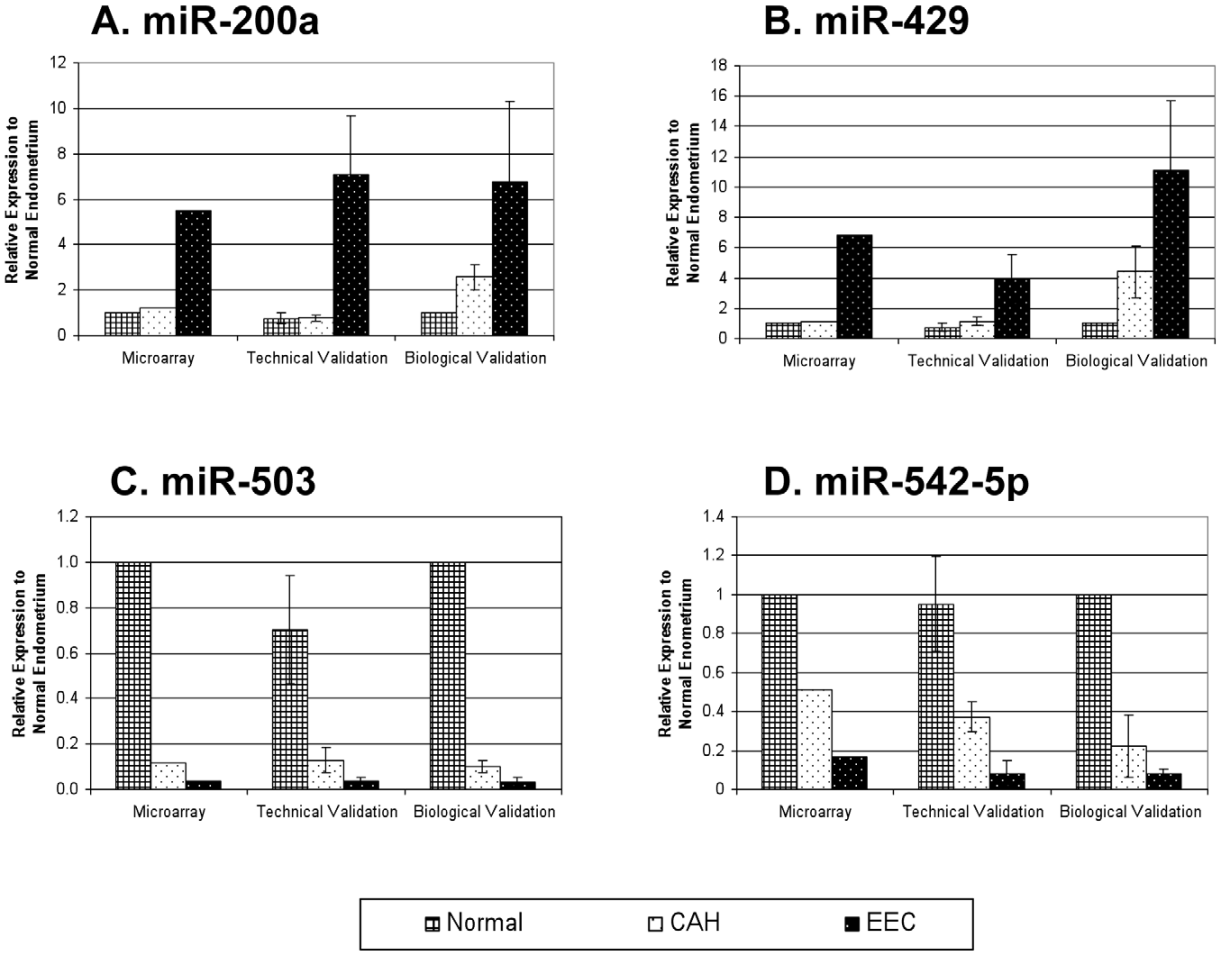
Quantitative RT-PCR results. Validation RT-PCR was performed using formalin-fixed paraffin embedded tissues from the original sample set as well as a new sample set using primers for miR-200a (A), miR-429 (B), miR-503 (C), and miR-542-5p (D). Error bars represent standard deviation.

The choice of miRNAs for validation with real-time RT-PCR was based on their differential expression among the groups as well as their putative biological significance: miR-542-5p is predicted to target estrogen receptor alpha mRNA; miR-200a and miR-429 are both members of the miR-200 family; and miR-503 is the most down-regulated miRNA in both types of carcinoma and the precursor lesion. Figure 2 depicts the results of the validation real-time RT-PCR. For all four of the miRNAs examined, changes in the expression pattern observed in the technical and biological validation sets were consistent with the changes observed by microarray.

## Discussion

The identification of molecular species that can differentiate between tumour and normal tissue, or between precursor and tumour tissues, is widely considered to be useful for a number of reasons. First, the identification of consistently dysregulated patterns of expression can lead to clues about the biological processes that lead to the development or progression of cancer. Second, the identification of these markers can provide rapid methods for diagnostic or prognostic tests. And thirdly, continued understanding of the pathways that are targeted during tumour development can ultimately lead to the identification of novel and specific druggable targets, ultimately affecting patient outcomes.

In the case of endometrial carcinoma, we set out to identify miRNA species that might differentiate between EEC and CAH, and differentiate both from normal controls. We argued that examination of the dysregulated miRNAs might reveal important steps in both early endometrial carcinogenesis and progression to invasive carcinoma. Additionally, a subset of the miRNAs show discordant expression in CAH and EEC compared to controls (ie: increased expression in CAH and decreased expression in EEC compared to controls; Table 2). There is the potential to use such miRNAs to distinguish between CAH and EEC which would be beneficial, especially on small biopsies where more complex testing might not be possible.

We identified 43 microRNAs that are dysregulated in EEC and CAH compared to normal controls. Clustering analysis shows that these 43 miRNAs can differentiate EEC from both CAH and normal controls. Furthermore, the dysregulated miRNAs show intermediate expression level changes in the precursor lesion, CAH.

Our data share some important similarities with previously published reports [22]–[26]; Table 4. The most striking similarity is the up-regulation of the miR-200 family (miR-141, 200a, 200b, 200c, 429) in EEC relative to normal controls. This up-regulation among different studies is observed consistently despite the use of different patient populations, different tissue types (fresh vs formalin-fixed), different investigative platforms (RT-PCR vs microarray) and different control tissues (atrophic vs proliferative endometrium vs mixed proliferative/secretory vs normal adjacent). In our study, this up-regulation is additionally observed in the precursor lesion.

**Table 4:** Summary of available studies detailing miRNA expression profiles in EEC compared to normal controls.

Study	Increased Expression	Decreased Expression	Tissues(Fresh unless specified)	Platform Used	Statistics
Boren et al 2008 [22]	let-7c *miR-103 miR-106a miR107* miR-181a miR-185 *miR-210 miR-423*	let 7i miR-152 miR-193 miR-221 miR-30c	Normal postmenopausal endometrium (n = 20)Atypical hyperplasia (n = 4)EEC (n = 37)	Microarray	Mann-Whitney, *p*<0.02
Chung et al 2008 [23]	*miR-103 miR-106a miR-107* *miR-10a* miR-130b ***miR-141***miR-151 *miR-155* miR-17-5p *miR-182 miR-183* miR-184 miR-191miR-194 ***miR-200a*** ** ***miR-200c*** * miR-203* *miR-205 miR-210* miR-215 *miR-223* miR-23a* miR-25miR-28 miR-301 miR-325 miR-326 miR-330 *miR-34a* miR-95		Normal endometrium (proliferative n = 7, secretory n = 7, postmenopausal n = 8)EEC (n = 30)	RT-PCR	T-test *p*<0.01, FC>2 with a mean difference of 100, FDR = 0.5%
Wu et al 2009 [24]	*miR-10a * ***miR-141*** miR-142-5p *miR-155 miR-182 * ***miR-200a*** ** ***miR-200b*** ** ***miR-200c*** * miR-203 miR-205 miR-210* miR-31 miR-363 ***miR-429*** miR-432 miR-449 miR-96	miR-133b miR-193a miR-193b miR-204 miR-368 miR-99b	EEC with normal adjacent endometrium (n = 10)	Microarray	SAM with FDR 0% and FC>3.0
Ratner et al 2010 [25]	*miR-182 miR-183 * ***miR-200a*** * miR-205 miR-34a* miR-572 miR-622 miR-650	miR-411 miR-487b	EEC (11 fresh and 46 FFPE) and 5 “benign endometrial tissues”	Microarray	T-test *p*<0.05
Cohn et al 2010 [26]	miR-146 miR-181c *miR-183* miR-19b ***miR-200c*** * miR-205 miR-223 miR-423* miR-425 miR-9	let-7a miR-32 miR-33b miR-369 miR-409 miR-424 miR-431 miR-451 miR-496 miR-503 miR-516	Stage 1 EEC (121 FFPE) and 20 normal unmatched endometrial samples (FFPE; 10 premenopausal and 10 postmenopausal)	Microarray	Class prediction method p<0.001

MicroRNAs which have similar expression changes among the studies are italicized and the miR-200 family miRNAs are bolded.

The miR-200 family consists of five members localized on two genomic clusters (miR-200a/b, miR-429 on chromosome 1, and miR-200c, miR-141 on chromosome 12) [27]. The miR-200 family has been implicated in the epithelial-to-mesenchymal transition (EMT) that occurs as a part of tumour invasion and metastasis [28]–[30]. Briefly, these miRNAs have been shown to negatively regulate two transcription factors, ZEB1 and ZEB2, which in turn negatively regulate E-cadherin. When ZEB1 and ZEB2 are overexpressed, levels of E-cadherin go down and cells adopt a mesenchymal phenotype that is better able to invade tissues and metastasize. Conversely, if ZEB1 and ZEB2 levels are low, as would be expected with high miR-205 and miR-200 family members, E-cadherin levels increase and an epithelial phenotype should be maintained.

Studies have shown an association with low miR-200 family levels and an aggressive tumour phenotype [31], [32] consistent with the regulation of the EMT switch as described. However, there are some tumour types in which an up-regulation of the miR-200 family has been observed. These include melanoma [33], [34], ovarian carcinoma [35] and colorectal carcinoma [36]. Cochrane et al [37] have shown that miR-200c levels are associated with a less aggressive tumour phenotype in ovarian cancer, breast cancer and EEC cancer cell lines. Recently, Castilla et al [38] have demonstrated differential miRNA expression levels between the epithelial and mesenchymal elements in uterine carcinosarcoma, with up-regulation of the miR-200 family in the epithelial part.

Insights into the up-regulation of the miR-200 family in certain tumour types, including endometrial carcinoma, may be gained by examining the role of steroid receptors in the regulation of miRNAs. A recent review by Cochrane et al [39] describes the regulation of the miR-200 family by estrogen receptor-α and estradiol. Endometrial carcinomas that express ERα tend to be less aggressive than tumours that have no ERα expression [40]. In the studies of miRNA expression in endometrial carcinomas, the up-regulation of the miR-200 family may be influenced by estradiol and ERα and this possibility deserves further study. It may be that the less aggressive nature of ERα-positive endometrial carcinomas relates to the up-regulation of the miR-200 family, which, in turn, maintains an epithelial phenotype and resists the EMT transition. In this study, there was no significant difference in miR-200 family levels between lower grade 1 and 2 tumours compared to grade 3 tumours (data not shown); however, this study did not have a sufficiently large sample size to see such a difference.

MicroRNAs are becoming important molecules in cancer research; they have been implicated in tumorigenesis and have been used both as biomarkers for cancer and as markers for prognostication. Previous studies have shown that miRNAs are dysregulated in EEC and this study identifies a similar miRNA expression profile. The most striking similarity among studies of miRNA expression in EEC is the up-regulation of the miR-200 family. While most tumours have shown a down-regulation of the miR-200 family, the up-regulation observed in endometrial carcinoma is shared with a few other tumours including melanoma, ovarian carcinoma, and colorectal carcinoma. Efforts to elucidate the role that miRNAs play in endometrial carcinoma should focus on the miR-200 family as well as the other miRNAs that are consistently dysregulated in the multiple studies of endometrial carcinoma to date.
